# Clinical Charateristics and Outcome of Pyogenic Liver Abscess with Different Size: 15-Year Experience from a Single Center

**DOI:** 10.1038/srep35890

**Published:** 2016-10-24

**Authors:** Zhao-Qing Du, Li-Na Zhang, Qiang Lu, Yi-Fan Ren, Yi Lv, Xue-Min Liu, Xu-Feng Zhang

**Affiliations:** 1 Department of Hepatobiliary Surgery, the First Affiliated Hospital Xi’an Jiaotong University, Xi’an, 710061, Shaanxi Province, China; 2 Institute of Advanced Surgical Technology and Engineering, the First Affiliated Hospital of Xi’an Jiaotong University, Xi’an, 710061, Shaanxi Province, China; 3 Shaanxi Provincial Regenerative Medicine and Surgical Engineering Research Center Xi’an, 710061, Shaanxi Province, China; 4 Department of Pharmacy, the Second Affiliated Hospital of Xi’an Jiaotong University, Xi’an, 710004, Shaanxi Province, China

## Abstract

The standard therapeutic protocols of pyogenic liver abscess (PLA) have not been well established yet. We investigated the clinical characteristics, disease progression, choices of treatments and outcomes of PLA with different size. 410 cases of patients with PLA were enrolled retrospectively from 2000 to 2014, and were grouped as small abscess (≤5 cm, n = 125), large abscess (5 cm to 10 cm, n = 218) and giant abscess (>10 cm, n = 36). The most common bacteria were *Klebsiella pneumonia* (22%) and *Escherichia coli* (11%) by pus culture, and *Escherichia coli* (36.7%), *gram-positive coccus,*(36.7%) and *Klebsiella pneumonia* (33.3%) by blood culture. 115 patients (28.0%) received antibiotics treatment alone, 161 patients (39.3%) received percutaneous drainage (PD) and 134 patients (32.7%) underwent surgical incision and drainage (SD). The size of abscess was correlated with leukocytes increase, albumin decrease, and time duration for body temperature normalization (all *p* < 0.05). Antibiotics treatment alone, PD and SD was mainly used in patients with small abscess (42.4%), large abscess (44.0%) and giant abscess (47.2%), respectively. For patients with giant abscess, SD group (n = 17) had higher morbidity than PD group (n = 14) (76.4% vs. 35.7%, *p* = 0.022). PD might achieve the same curative rate as SD in giant abscess, but with less trauma, lower morbidity and shorter hospital stay.

Pyogenic liver abscess (PLA) is a potentially life-threatening condition of hepatobiliary system infection, with mortality up to 19%^1^,^2^,^3^. The incidence of PLA was reported greatly different among general population, as ranging from 1.1–3.6 per 100 000 population in European and America, but as high as 17.6 per 100 000 population in Asia^3^,^4^. However, the mortality of PLA has been dramatically decreased in experienced centers due to earlier and improved diagnosis, development of intensive care, and progression of minimal invasive treatment^1^.

Intravenous use of antibiotics is considered as one of the most critical measure for treatment of PLA. Use of antibiotics is mostly effective in controlling symptoms of patients with small liver abscess. However, for large liver abscess, single use of antibiotics is insufficient due to higher bacterial load, inadequate penetration of antibiotics and ineffective medium for bacterial elimination^1^,^5^. Effective drainage is recognized as the most effective treatment for large liver abscess, since it could definitely lower bacterial load and increase antibiotics penetration into the abscess^6^. Percutaneous drainage (PD) and surgical incision and drainage (SD) are two mainstays of abscess drainage. However, there was much debate on the abscess size and choice of drainage procedures. Many surgeons advocated use of PD in abscess around 3–6 cm, and SD in abscess with larger size for complete drainage^7^,^8^,^9^. Although previous study has shown that abscess size greater than 7.3 cm predicted failure of PD, a recent study demonstrated that abscess greater than 10 cm could be successfully treated with PD, with a failure rate of 2.6%^1^. Unfortunately, there was no parallel control group in this study to evaluate the successful rate and complications between PD and SD in giant PLA. Therefore, the present study aims at evaluating three different treatments (e.g. antibiotics only, PD and SD) in abscess with different size during a 15-year period from the largest academic center and university affiliated hospital in Northwest China.

## Results

Totally, 410 patients were enrolled into the present study from 2000 to 2014 in our hospital. As summarized in Table 1, the median age was 57 years old (range: 1–89 years) with predominantly male (61.5%). 116 (28.3%) patients had concurrent diabetes. 89 (21.7%) patients had recent respiratory or enteric infection before admission. Totally, 141 (34.4%) patients had hepatobiliary surgeries, and four (1%) patients had surgical incision and drainage of liver abscess previously, the details of which were documented in Table 1. Fever was the most common symptoms (91.5%), and then abdominal pain (56.8%). Jaundice was not common and only presented in seven patients (1.7%).

As presented in Table 2, leucocytes were abnormally elevated in 187 patients (45.6%) at admission to our hospital. And most patients (228/410, 55.6%) had normal body temperature below 37.3 °C at admission, while only 31 (7.5%) patients presented with high fever (above 39.1 °C). The median time for normalization of the body temperature was seven days (range: 0–67 days), and 21 patients had abdominal high temperature when discharged and treated consecutively in local hospital till control of the symptoms. Most patients had solitary abscess (274/410, 66.8%), and 122 (29.7%) had two or more abscess in liver. There were 125 (30.5%) patients with small abscess (≤5 cm), 218 (53.2%) patients with large abscess (5–10 cm) and 36 (8.8%) patients with giant abscess (>10 cm). Pus culture was available in 256 (62.4%) patients. *Klebsiella pneumonia* (22%) and *Escherichia coli* (11%) were the most two common pathogen. While 208 (81.3%) showed positive in pus culture, 48 (18.7%) patients showed negative in pus culture. Blood cultures were available in 119 (29%) patients. 30 of the 119 patients (25.2%) with available data presented with positive bacterial culture, and *Escherichia coli* (11/30, 36.7%), *Gram-positive coccus* (11/30, 36.7%) and *Klebsiella pneumonia* (10/30, 33.3%) were the most pathogens cultured.

161 (39.3%) patients underwent PD, 134 (32.7%) patients received SD, and the rest 115 (28%) patients were administrated intravenous antibiotics treatment alone. Most patients (408/410, 99.5%) were cured, while only two (0.5%) patients died in hospital stay. One died of severe general sepsis and the other one died of cachexia and finally multiple organ failure.

We categorized all the 379 patients with intact information by the size of the abscess as small, large and giant lesions. The clinical manifestation, laboratory tests, treatments and outcomes were compared among the three groups (Table 3). There was no significant difference in gender, age, comorbidity, surgical history, or abscess numbers between the three groups. However, patients with larger abscess exhibited significantly higher white blood cell count but lower albumin level (*p* = 0.013, and *p* = 0.002, respectively, Table 3). The time duration for body temperature normalization after admission was longer in patients with giant abscess than those with large or small abscess (*p* = 0.005, Table 3). The choices of therapeutic regimen were differently distributed among the three groups (*p* < 0.001). Conservative treatments with antibiotics alone was mainly adopted in patients with small abscess (42.4%), PD was mainly performed in patients with large abscess (44.0%) and SD was mainly performed in patients with giant abscess (47.2%). Although the outcomes of the patients were similar among the three groups, patients with giant abscess had significantly longer hospital stay than other two groups (median time, 23 days vs. 18 days and 18 days, *p* = 0.003).

The effects and outcomes of PD (n = 14) and SD (n = 17) in patients with giant abscess were further compared (Table 4). There was no difference between the two groups in age, abscess number, presence of cavity separation, leukocytes count, liver function, etc. However, the incidence of complications in SD group was two times higher than that of PD group (76.4% vs. 35.7%, *p* = 0.022, Table 4). It was documented that patients in SD group had higher incidence of severe complications (bile leakage, abdominal bleeding, pulmonary infection and pleural effusion necessitating drainage) than PD group, the difference of which was, however, not statistically different (Table 4). Due to falling off of the drainage tube, three cases in PD group experienced replacement of the drainage tube. All the patients in the two groups were cured. However, SD group had longer hospital stay than PD group (median time, 29 days vs. 12 days, *p* = 0.024).

## Discussion

The etiology of liver abscess mainly includes infection of liver through portal vein, retrograde infection through biliary, systemic blood infection and other unknown occult infections^10^. And the incidence of PLA in patients with diabetes, hepatobiliary diseases, pancreatic diseases, abdominal infection, and hepatic or biliary tract surgery history was higher than that of common individuals^11^. Among our patients cohort, 28.3% had diabetes and 21.7% had documented respiratory or gastrointestinal infection history, implying that impairment of immune function and systemic infection might induce liver abscess formation via hematogenous dissemination. In addition, 34.4% of the patients with unambiguous abdominal surgery history might be related to functional changes of the biliary tract, such as intestinal juice reflux after choledochojejunostomy and duodenal papilla sphincter dysfunction after cholecystectomy, which might cause retrograde infection of liver parenchyma^12^. Due to severely systemic infection, fever was the most common clinical symptom (91.5%), followed by abdominal pain (56.8%) as a result of enlargement of abscess and tension of hepatic capsule. Most patients showed elevated leukocyte levels, but those with severe infection might present myelotoxicity with low leukocyte, platelets, or hemoglobin levels^6^,^13^. Abdominal computer tomography (CT) and ultrasound examination for liver abscess patients had a high accuracy up to 90%, and simultaneously the etiology might be ascertained. For this reason, abdominal CT and ultrasound was recommended as routine examinations^9^.

In the present study, the positive rates of bacterial culture in pus and blood were 81.3% (208/256) and 25.2% (30/119), respectively. These findings implied that sepsis was mostly confined to liver. However, administration of antibiotics and anti-fever drugs might significantly lower the positive rate of blood culture. Notably, *Klebsiella pneumoniae* turned into the most common causes of liver abscess in our study other than E.coli, which is consistent with the recent findings from other studies from Asian contries, implying a gradual change of the bacterial spectrum of liver abscess^14^. It might be confirmed that extrahepatic and then systemic infection has gradually become the primary causes for liver abscess, especially in patients with diabetes mellitus^14^,^15^,^16^. It has been found that *E. coli* PLA was more commonly associated with hepatobiliary disease, while *Klebsiella pneumoniae* PLA were larger in size and had more invasive treatments. However, the median hospital stay or outcomes seemed not to be different between two different PLA^17^,^18^. Interestingly, it has been reported recently that culture negative PLA had the similar outcome to *Klebsiella Pneumoniae* PLA^19^.

The treatments of liver abscess are mainly based on the personal experience of clinicians with the lack of uniform standards. To lower bacterial load and control the symptoms of systemic infections, antibiotic therapy has been recommended to use at the early stage of the disease^13^,^20^. It has been reported that for liver abscess less than 5 cm in diameter, antibiotics alone could reach cure rate higher than 80%^6^. In our study, 42.4% (53/125) patients with small abscess were treated with antibiotics alone, and the effective rate was 100%. Accordingly, antibiotics used empirically should cover *Gram-negative bacilli* and *Gram-positive coccus*, such as combining the antibiotic of the third cephalosporin with aminoglycoside or metronidazole^1^,^7^,^20^. And adjustment of antibiotics might be necessary according to bacterial culture and drug sensitivity. Moreover, the use of antibiotics should be as long as 2–6 weeks^1^,^7^,^20^. In some complicated cases, antimicrobial therapy is usually continued after source control^21^. However, the growing emergence of multi-drug resistant organisms and the limited development of new agents to counteract them have caused an impending crisis, especially with regards to Gram-negative bacteria^21^.

Due to minimal injury, accurate drainage, and rapid recovery of patients, PD has become the uppermost remedy of liver abscess. The traditional SD has been relegated to second-line treatment. However, PD is still subject to many restrictions, such as with or without completely abscess liquefaction and too many separation of the pus. At present, there is still a great controversy on the relationship between the two types of treatments and the abscess size. Liao *et al*. proposed that abscess diameter larger than 7.3 cm may indicate poor puncture drainage, and therefore should be considered for surgical incision and drainage^22^. Tan *et al*. reported that for abscess larger than 5 cm, surgical incision had a higher success rate, lower reoperation, and a shorter hospital stay, and should be recommended^9^. However, the latest study from Ahmed confirmed that on account of effective antibiotics, even for the giant abscess larger than 10 cm, curative ratio of PD reached 97.4% and failure rate or re-puncture drainage rate were as low as 2.6% and 7.7%^1^. The study also affirmed the abscess size was positively correlated with the severity of the diseases^1^. In our study, for giant abscess, complications in SD group was two times as much as that of PD group (76.4% vs. 35.7%, *p* = 0.022). PD induced shorter hospital stay than SD group (median time, 29 days vs. 12 days, *p* = 0.024), although all the patients in the two groups were cured. These retrospective results prompted that even for giant abscess, PD could achieve the same curative ratio as SD, but less trauma, lower complication rates and shorter hospital stay. Therefore, abscess size should not be used as the indication of puncture drainage or incision. In the case of failure or inefficiency of PD, multiple abscess, or palpable purulent cavity, timely SD could achieve effective drainage. The 30-day mortality rate for liver abscess was reported ranging from 0% to 17% from different studies^1^,^9^,^10^,^23^,^24^. Only two patients (0.5%) died during hospital stay, while the rest of them were finally cured. The lower 30-day mortality might be attributed to the early diagnosis and effective treatments.

In conclusion, hematogenous dissemination and retrograde infection via biliary tract are still the primary reasons for the liver abscess generation. Meanwhile, *Klebsiella pneumoniae* has turned into the most common pathogenic bacteria in liver abscess. For giant hepatic abscess, PD may achieve the same curative ratio as SD, but less trauma, lower morbidity and shorter hospital stay. Therefore, PD should be firstly recommended in large and giant abscess (larger than 5 cm in diameter).

## Patients and Methods

A retrospective review of medical record database was undertaken for patients diagnosed primarily as “Liver abscess” between January 1, 2000 and December 31, 2014 in the First Affiliated Hospital of Xi’an Jiaotong University. Five cases with ameboid liver abscess had been excluded from the study. Totally, data was available for 410 consecutive patients. The study was approved by the ethics committee of the First Affiliated Hospital, Xi’an Jiaotong University the premise of obtaining the voluntary informed consent of enrolled patients, the rules of which comply with the Treaty of Helsinki. A waiver of informed consent was obtained, since the data were analyzed from the electronic medical record and reported without personal identifiers.

The methods were carried out in accordance with the approved guidelines from he ethics committee of the First Affiliated Hospital, Xi’an Jiaotong University. Clinical data was documented by retrospective chart review. Patient variables included age, gender, comorbidities, prodroma, surgery history, symptoms and body temperatures at admission and changes during hospitalization. Laboratory values included blood routine test values and liver function, which were documented at admission. Abscess number and size were evaluated based on radiological images and/or laparotomy. Abscess were classified as small abscess (≤5 cm in maximal diameter), large abscess (5–10 cm in maximal diameter) and giant abscess (>10 cm in maximal diameter).

All the patients, once diagnosed with PLA, were usually treated by a combination of a two or third-generation cephalosporin and metronidazole empirically or based on identification of bacteria and susceptibility test if available. Usually Treatments of PLA (antibiotics alone, PD or SD) were decided according to abscess size, number, degree of abscess liquefaction, separation of abscess cavity, patient response to antibiotics and personal experience of the physicians. All the patients with positive blood culture were treated with intravenous antibiotics for 7 to 14 days or even longer depending on the clinical and radiological response.

For patients undergoing PD, a 12 to 14 Fr drains were placed into the abscess cavity monitored by ultrasound. And the volume of drains was documented daily. Once the volume of drains was decreased less than 10 ml/day, the patients would undergo ultrasound examination for confirmation of drains displacement, obstruction or complete drainage. The drainage tube would then be repositioned, replaced or withdrawn depending on imaging findings. SD was mostly performed for patients with large or giant abscess with thick pus, multiple abscess separation, failure of PD due to location or ineffective drainage, or abscess rupture. Abscess cavity was determined with the help of ultrasound. Abscess wall was deroofed, and loculations were broken down. For patients with concurrent biliary disorders, additional biliary tract procedures were performed. Liver resection was performed for abscess with complete destruction of a segment or section of liver parenchyma.

### Statistical analysis

Numerical variables were expressed as median and range, while categorical variables were expressed as number of events and percentage. U or t test was used for comparing the numerical variables between two groups, and variance analysis was used for comparing numerical variables among three or more groups. The categorical variables were analyzed by pearson Chi square test or Fisher’s exact test. *p* < 0.05 were considered statistically different.

## Additional Information

**How to cite this article**: Du, Z.-Q. *et al*. Clinical Charateristics and Outcome of Pyogenic Liver Abscess with Different Size: 15-Year Experience from a Single Center. *Sci. Rep.* **6**, 35890; doi: 10.1038/srep35890 (2016).

## Figures and Tables

**Table 1:** Characteristics of the 410 patients with pyogenic liver abscess presented at admission.

Clinical charateristics	Median (range)/*n* (percentage)
Age (years)	57 (1–89)
Gender (male:female)	252:158
Diabetes mellitus	116 (28.3%)
Prodroma	
Respiratory tract infection	82 (20%)
Enteric infection	7 (1.7%)
Surgery history	141 (34.4%)
Liver abscess incision and drainage	4 (1.0%)
Cholecystectomy	34 (8.3%)
Common bile duct exploration	19 (4.6%)
Biliary-enteric anastomosis	45 (11.0%)
Pancreaticoduodenectomy	10 (2.0%)
Liver transplantation	7 (1.7%)
Liver resection	10 (2.4%)
Alimentary tract surgery	8 (2.0%)
Liver cancer radiofrequency ablation	3 (0.7%)
Liver cancer interventional therapy	2 (0.5%)
Other surgery	1 (0.2%)
Symptoms	
Fever	375 (91.5%)
Abdominal pain	233 (56.8%)
Jaundice	7 (1.7%)

**Table 2:** Laboratory tests, clinical characteristics, treatments and outcomes of the patients.

Clinical charateristics	Median (range)/*n* (percentage)
Laboratory tests	
Leucocytes >10 × 109/L	187 (45.6%)
Hemoglobin <120 g/L	271 (66.1%)
Platelet count <100 × 10^9^/L	69 (16.8%)
Alanine transaminase >40 U/L	189 (46.1%)
Aspartate transaminase >40 U/L	151 (36.8%)
Total bilirubin >17 μmol/L	151 (36.8%)
Albumin <35 g/L	304 (74.1%)
Body temperature at admission (°C)	37.4 ± 1.1
35.5 °C–37.3 °C	228 (55.6%)
37.4 °C–39 °C	151 (36.8%)
>39.1 °C	31 (7.5%)
Time for temperature normalization (days)*	7 (0–67)
Abscess number**	
Solitary abscess	274 (66.8%)
Multiloculation	122 (29.7%)
Maximal diameter of abscess***	
≤5 cm	125 (30.5%)
5–10 cm	218 (53.2%)
>10 cm	36 (8.8%)
Pus culture****	
*Klebsiella pneumonia*	90 (22.0%)
*Streptococcus spp*	16 (3.9%)
*Staphylococcus spp*	15 (3.7%)
*Escherichia coli*	45 (11.0%)
*Enterococcus spp*	21 (5.1%)
*Bacteroides spp*	13 (3.2%)
*Fungus*	8 (2.0%)
*Negative*	48 (11.7%)
*Unavailable*	154 (37.6%)
Blood culture****	
*Klebsiella pneumonia*	10 (2.4%)
*Escherichia coli*	11 (2.7%)
*Gram-positive coccus*	11 (2.7%)
*Gram-positive bacillus*	3 (0.7%)
*Fungus*	1 (0.2%)
*Negative*	89 (21.7%)
*Unavailable*	291 (71.0%)
Treatment	
Percutaneous drainage	161 (39.3%)
Surgical fenestration and drainage	134 (32.7%)
Conservative treatment	115 (28.0%)
Outcomes	
Cured	408 (99.5%)
Died in hospital	2 (0.5%)
Hospital stay (days)	18 (2–71)

^*^21 patients had abnormal body temperature when discharged.

^**^Data of 14 patients were missed.

^***^Data of 31 patients were missed.

^****^Some patients had more than one bacteria in cultures.

**Table 3:** Clinical presentation and outcome of the 379 patients with different size of abscess.

Variables	Small abscess (≤5 cm)	Large abscess (5–10 cm)	Giant abscess (>10 cm)	*p* value
*n*	125	218	36	
Gender (male)	76 (60.8%)	135 (61.9%)	20 (55.6%)	0.768
Age	56 (2–85)	58 (1–89)	59 (13–78)	0.294
Diabetes mellitus	30 (24%)	65 (29.8%)	9 (25.0%)	0.480
Prodroma				0.071
Respiratory infection	21 (16.8%)	55 (25.2%)	5 (13.9%)	
Enteric infection	0	6 (2.8%)	1 (2.8%)	
Biliary surgery	37 (29.6%)	70 (32.1%)	8 (22.2%)	0.466
Solitary abscess	81 (64.8%)	159 (72.9%)	27 (75.0%)	0.490
Leucocytes >10 × 10^9^/L	46 (36.8%)	108 (49.5%)	22 (61.1%)	0.013
Hemoglobin <120 g/L	79 (63.2%)	147 (67.4%)	27 (75.0%)	0.385
Platelet count <100 × 10^9^/L	17 (13.6%)	38 (17.4%)	6 (16.7%)	0.636
Alanine transaminase >40 U/L	52 (41.6%)	104 (47.7%)	16 (44.4%)	0.665
Aspartate transaminase >40 U/L	43 (34.4%)	76 (34.9%)	18 (50.0%)	0.157
Total bilirubin >17 μmol/L	41 (32.8%)	80 (36.7%)	18 (50.0%)	0.138
Albumin <35 g/L	81 (64.8%)	172 (78.9%)	31 (86.1%)	0.002
High temperature at admission (>37.3 °C)	49 (39.2%)	99 (45.4%)	20 (55.6%)	0.209
Time for temperature normalization (days)	7 (0–67)	7 (0–36)	12 (0–36)	0.005
Treatment				<0.001
Conservative treatment	53 (42.4%)	6 (2.8%)	5 (13.9%)	
Percutaneous drainage	35 (28.0%)	96 (44.0%)	14 (38.9%)	
Surgical drainage	37 (29.6%)	76 (34.9%)	17 (47.2%)	
Outcomes				0.125
Cured	125 (100%)	217 (99.5%)	35 (97.2%)	
Died in hospital	0	1 (0.5%)	1 (2.8%)	
Hospital stay (days)	18 (3–71)	18 (2–57)	23 (4–61)	0.003

**Table 4:** Comparison of percutaneous drainage and surgical incision and drainage in giant abscess.

Variables	Percutaneous drainage (n = 14)	Surgical incision and drainage (n = 17)	P value
Age (years)	58 (28–78)	59 (13–77)	0.959
Multiple abscess (≥2)	4 (28.6%)	3 (17.6%)	0.469
Abscess cavity seperation	6 (42.9%)	9 (52.9%)	0.576
Leucocytes >10 × 10^9^/L	10(71.4%)	10(58.8%)	0.707
Hemoglobin <120 g/L	11(78.6%)	12(70.6%)	0.698
Platelet count <100 × 10^9^/L	2(14.3%)	2(11.8%)	1.000
Alanine transaminase >40 U/L	5(35.7%)	8(47.1%)	0.484
Aspartate transaminase >40 U/L	6(42.9%)	9(52.9%)	0.715
Total bilirubin >17 μmol/L	8(57.1%)	8(47.1%)	0.730
Albumin <35 g/L	13 (92.9%)	15 (88.2%)	1.000
Complications	5 (35.7%)	13 (76.4%)	0.022
Bile leakage	0	3 (17.6%)	0.098
Intraperitoneal bleeding	1 (7.1%)	4 (23.5%)	0.217
Pulmonary infection	1 (7.1%)	2 (11.8%)	0.665
Pleural effusion with drainage	3 (21.4%)	7 (41.2%)	0.242
Re–treatments	3 (21.4%)	0	0.045
Hospital stay (days)	12 (4–36)	29 (14–61)	0.024
Cured	14 (100%)	17 (100%)	1.000
